# Validating the evaluation capacity scale among practitioners in non-governmental organizations

**DOI:** 10.3389/fpsyg.2022.1082313

**Published:** 2022-12-23

**Authors:** Steven Sek-yum Ngai, Chau-kiu Cheung, Yunjun Li, Lifen Zhao, Lin Wang, Shan Jiang, Hon-yin Tang, Elly Nga-hin Yu

**Affiliations:** ^1^Department of Social Work, The Chinese University of Hong Kong, Shatin, Hong Kong SAR, China; ^2^Department of Social and Behavioral Sciences, City University of Hong Kong, Kowloon, Hong Kong SAR, China; ^3^Department of Social Security, Nanjing Normal University, Nanjing, China; ^4^Department of Social Work, Fudan University, Shanghai, China; ^5^Department of Sociology, Zhejiang University, Hangzhou, China

**Keywords:** non-governmental organization, internal evaluation, assessment of evaluation capacity, scale validation, Hong Kong

## Abstract

The growing emphasis on demonstrating the effectiveness of social services through evaluation has heightened demand for nongovernmental organization (NGO) practitioners to enhance evaluation capacity. However, a lack of validated instruments in the NGO context has hampered efforts to assess NGO practitioners’ current evaluation capacity and understand how capacity-building activities could be tailored to meet NGO practitioners’ actual needs and enhance their evaluation capacity. Hence, this study aims to develop the Evaluation Capacity Scale (ECS), a self-reporting instrument of NGO practitioners’ capacity to conduct an effective evaluation of their service programs. Validation data was derived from 439 NGO practitioners who attended the Jockey Club MEL Institute Project in Hong Kong, China. Exploratory factor analysis of the ECS revealed three factors—evaluation mindset, evaluation implementation, and evaluation communication—and confirmatory factor analysis further validated this three-factor structure. Moreover, MANCOVA analysis demonstrated the ECS’s predictive validity. Overall, the ECS demonstrated satisfactory convergent validity, high internal consistency reliability, and predictive validity, and its factor structure was supported in subgroups based on gender, age, and level of education. Theoretical and practical implications of the findings are discussed.

## 1. Introduction

The increased importance and visibility of non-governmental organizations (NGOs) addressing social problems have heightened demand for greater transparency and accountability for the effectiveness and societal impact of various NGO programs (Suárez and Marshall, 2012; Doherty et al., 2015). In an era when cost-effectiveness is paramount, NGOs are under pressure to improve program performance and demonstrate the effectiveness thereof (Marshall and Suárez, 2013). Due to the growing trend of contracting out government services in the past two decades, policy makers need to know the quality and value of specific public services for funding-related decisions (Spolander et al., 2014; Szczepanska, 2020). Likewise, NGO funding agencies ask for evidence to verify whether social service programs achieve specific outcomes and request grantees to regularly report performance-related information (Humphries et al., 2010; D’Ostie-Racine et al., 2016). Due to pressure from policy makers and funding agencies, NGOs have stressed the importance of learning about the effectiveness of their services so as to become more accountable and professional (Suárez and Marshall, 2012; Chauveron et al., 2021).

In response to the preceding demands for systematic evidence about the effectiveness of social services, the ability of NGO practitioners to conduct internal evaluations—evaluating service programs by themselves within their own organizations, instead of contracting external evaluation consultants to carry out evaluations—is an important topic of study in this field (Marshall and Suárez, 2013; Kelly and Rogers, 2022). By definition, evaluation refers to a broad range of activities undertaken by NGOs to assess their organization’s performance, improve the effectiveness of their programs, and meet the needs of diverse stakeholders (Marshall and Suárez, 2013; Tarsilla, 2014). Several studies have found the internal evaluation activities of NGOs have intensified (Cousins et al., 2008; Clinton, 2014; Kelly and Rogers, 2022). During the past two decades, significant effort was invested in building the capacity of NGO practitioners (internal evaluators) to carry out effective evaluations themselves (Love, 1991; Taylor-Ritzler et al., 2013; Szczepanska, 2020). Despite increasing recognition of the demand to understand the evaluation capacity, few tools are available for NGO leaders, NGO practitioners, and researchers to assess the evaluation capacity (EC) of NGO practitioners (Nielsen et al., 2011). This has further hampered efforts to investigate how evaluation capacity-building (ECB) activities could find specific pain points, as well as tailor-training focus for NGO practitioners to meet their actual needs and enhance their EC (Suarez-Balcazar and Taylor-Ritzler, 2013).

As in many other parts of the world, the NGOs in Hong Kong have experienced increased accountability pressure from policy makers and funding agencies to prove their effectiveness in recent years. In 2000, influenced by the neoliberal ideology driving globalization, Hong Kong’s policy makers began to use lump-sum grant subventions *via* competitive bidding, which cultivated a contract-focused culture that emphasized monitoring output and outcomes (Nip, 2010). In this context, a growing need exists to increase the effectiveness and social impact of social service programs. According to the Hong Kong Jockey Club Charities Trust (2017), 76% of their grantees believed that monitoring, reporting, and/or carrying out evaluations added value to their programs. To that end, systematic monitoring and evaluation can be achieved in various ways. Whereas some NGOs have adequate resources—including the necessary funding and personnel to conduct research that supports evidence-based practices—other organizations invite investigators from universities or research institutes to perform external evaluations. Likewise, while Hong Kong NGO practitioners’ EC is potentially modifiable through engaging in capacity-building activities, one significant barrier to understanding their current capacity is the lack of validated instruments.

Hence, the current study aims to address this gap, through the development and validation of the Evaluation Capacity Scale (ECS), a rigorous self-reporting measure of NGO practitioners’ capacity to conduct evaluations. Validation data was derived from the Jockey Club MEL Institute Project (see the section on “The Present Study and Participants” for more details of the project), an ECB activity in Hong Kong that aims to build NGO practitioners’ EC by organizing a training and mentorship program.

## 2. Literature review

### 2.1. Significance of evaluation capacity for NGOs

In the NGO sector, conducting evaluations is a crucial approach to ensure that interventions are evidence-based and equitable, and service providers are more accountable for funds expenditures (Harman, 2019; Kelly, 2021; Kelly and Rogers, 2022). Generally, such evaluation consists of periodic assessments of the outcomes, efficiency, and impact of programs, with a specific focus on how the effects of a given program align with the expectations of the organization and the shareholders (Marshall and Suárez, 2013). More broadly, evaluation could be undertaken with an aim to gain applicable knowledge that can benefit the NGO sector (Mueller-Hirth, 2012). NGOs have long regarded evaluation as a means of gaging an organization’s accountability to funding agencies (Kelly and Rogers, 2022). Demonstrating accountability to funding agencies has become essential for survival in today’s competitive NGO sector (Cousins et al., 2014). Moreover, in addition to enabling NGOs to track program implementation and facilitating early identification of problems, effective evaluation practices could yield useful insights into specific evaluative practices and provide recommendations to improve NGO program planning and service delivery (Clinton, 2014; Kelly, 2021; Kelly and Rogers, 2022).

Unfortunately, the literature has extensively documented that NGO practitioners’ ability to carry out evaluations has not kept pace with this trend of increasingly valuing evaluation, including lacking evaluative knowledge or skills, holding negative attitudes toward evaluation activities, and being unfamiliar with evaluation procedures (Taylor-Ritzler et al., 2013; Kelly and Rogers, 2022). In this context, building NGO practitioners’ capacity to evaluate programs has become a major focus of overarching capacity-building programs in the NGO sector, particularly as a means of creating and sustaining organizational evaluation processes (Preskill and Russ-Eft, 2015). As such, an imperative need exists to develop a validated instrument that can be suitable for use by NGO leaders, NGO practitioners, and researchers to assess the status of NGO practitioners’ EC as well as to understand changes in these capacities following ECB activities.

### 2.2. Understanding evaluation capacity

In the evaluation literature, EC is a multidimensional concept with little consistency in how it is defined (Nielsen et al., 2011; Taylor-Ritzler et al., 2013; Morkel and Ramasobana, 2017; Kelly and Rogers, 2022). Approaching the issue from different focuses, researchers have identified numerous multidimensional competencies of NGO practitioners as necessary for effective evaluation practice, which collectively contribute to our understanding of EC’s definition.

First, many researchers have proposed that EC refers to not only the cognitive domain to have sufficient evaluation knowledge but also the affective domain to be aware of the importance of evaluation, as well as being willing to acquire and use evaluation knowledge and tools (Tarsilla, 2014; Harman, 2019; Chauveron et al., 2021). In this regard, Doherty et al. (2015) have summarized that the evaluation mindset—the capacity to understand evaluation and the readiness to use it for examining service effectiveness—is essential to embed evaluations as a domain of ongoing work within NGOs. Similarly, Bourgeois and Cousins (2013) referred to the evaluation mindset as NGO practitioners’ familiarity and interest in applying evaluation principles and practices. Moreover, prior research has also focused a great deal on the cultivation of an evaluation mindset among NGO practitioners as a key objective or outcome of ECB activities, including an awareness of the benefits of evaluations and the motivation to perform them, as well as the practitioners’ cognitive ability to engage in evaluation practices (Suarez-Balcazar and Taylor-Ritzler, 2013; Preskill and Russ-Eft, 2015; Morkel and Ramasobana, 2017).

Second, apart from the mindset domain, NGO practitioners’ practical ability to perform rigorous evaluations within their organizations is suggested as a fundamental indicator of understanding EC (Sonnichsen, 2000; Cousins et al., 2008; Preskill and Boyle, 2008; Taylor-Ritzler et al., 2013). For instance, many researchers have defined evaluation implementation as the practitioners’ behavioral ability to transfer learned evaluation knowledge and skills into organizational evaluation processes and practices (Taylor-Ritzler et al., 2013). In particular, evaluation implementation is often referred to as the practice of applying evaluative knowledge and skills at the organizational level, namely the capacity to do internal evaluations with a particular emphasis on the flow of the different phases of an evaluation (Cousins et al., 2008; García-Iriarte et al., 2010). Thus, evaluation implementation, as EC’s second domain, tends to emphasize knowledge translation process by taking action to apply evaluation knowledge and tools in daily practices (Preskill and Boyle, 2008; Preskill and Russ-Eft, 2015; Brown and Kelsey, 2016), including tracking program implementation, collecting process data, making internal refinements, and evaluating the final outcomes of the program (Bourgeois and Cousins, 2013; Kettner et al., 2016).

Third, since NGOs are increasingly expected to visualize their accountability and social impact *via* social media platforms, evaluation communication has recently been proposed as an important domain of EC (Medina et al., 2015; Kettner et al., 2016; DeCorby-Watson et al., 2018). For example, Mitchell (2017) defined evaluation communication as the ability of NGO practitioners to leverage communication channels and opportunities to collect and disseminate evaluation information. According to Preskill and Boyle (2008), such communication practices could significantly affect how people learn about evaluation and the extent to which evaluation practices are sustainable. Furthermore, the digital-tool boom that opened up new opportunities to disseminate the NGOs’ evaluation results should not be ignored. With the rapid advances in usage simplicity and the flexible convenience of digital tools, NGOs have been observed increasingly adopting the internet and various social media platforms for presenting their service effectiveness and impact to the public (Macnamara and Zerfass, 2012; Sinclair et al., 2016). Likewise, some researchers have emphasized that evaluation communication could be understood as a full range of learning about how to communicate the evaluation process and results to different stakeholders—e.g., service recipients, funding agencies, and the public—*via* different communication channels (e.g., digital tools) and at dissemination opportunities (e.g., press interviews, conferences; Kettner et al., 2016; Harman, 2019; Prosek, 2020).

Altogether, based on the extant evaluation literature, three distinct domains of EC have been identified for this study to assess NGO practitioners’ multidimensional competencies to conduct effective evaluation. These domains are (1) evaluation mindset, (2) evaluation implementation, and (3) evaluation communication. These three factors will be used to constitute our conceptual domain of EC since this three-factor framework reflects a perspective that could systematically and simultaneously delineate the multiple aspects of EC, which involves not only the cognitive and affective domains but also behavioral implementation and evaluation communication. With this multidimensional framework of EC, our study aims to create a measurement tool for systematically assessing the various domains of EC among NGO practitioners.

### 2.3. Existing measures of evaluation capacity

Despite the significance of NGOs implementing evaluations themselves, a limited selection of tools is available for measuring practitioners’ capacity to do so. The majority of the existing instruments are checklists developed after systematic analyses of the literature (Preskill and Boyle, 2008), which do not produce numeric scores to be correlated with other measures theoretically associated with EC, thus making it difficult to test construct validity with statistical analyses. This deficiency underscores the need for an empirical validation of evaluation scales. Moreover, instead of validated instruments, most existing assessment tools in the literature have been guidelines, intrinsically general and unable to accurately evaluate whether the ECB efforts are effective in enhancing NGO practitioners’ EC within NGOs (Bourgeois et al., 2018). A typical statement from Bourgeois et al.’s (2018) instrument asks respondents if they have the capacity to conduct evaluations in-house.

Existing measures generally come in a variety of lengths and are seldom designed for assessing EC covering all three domains—evaluation mindset, evaluation implementation, and evaluation communication. For instance, Arnold (2006) developed a seven-item scale and asked study participants to rate their overall EC, knowledge of evaluation, and competence in evaluation; notably, the items in this scale are very general, which makes it difficult to discern specific implications for future interventions. Similarly, Brandon and Higa (2004) developed and validated a five-item scale composed of general items (e.g., “*How important do you think program evaluation is?*”), and this instrument only assesses the affective domain of practitioners’ ability to perform evaluations, but does not include relevant items for evaluation practices. The most commonly used scale for EC is the 68-item Evaluation Capacity Assessment Instrument devised by Taylor-Ritzler et al. (2013), which measures NGO practitioners’ capacity to perform an evaluation and facilitates the use of the results to improve their abilities in this area. The length of this scale can be challenging for respondents, however, and even though the scale focuses on what practitioners think and how they implement evaluations, it does not include evaluation communication, an underexamined yet crucial domain of EC (Preskill and Boyle, 2008; Medina et al., 2015).

Overall, the lack of an empirical scale with operational items together with the constraints of extant scales due to limited domains highlights the need to develop a validated scale with operational items to cover various domains of EC. Hence, this study is meant to complement previously reported measures, with the aim of enabling more comprehensive assessment of the individual capacity to conduct an effective evaluation of their service programs among NGO practitioners.

## 3. Materials and methods

### 3.1. The present study and participants

Data for our study were derived from the Jockey Club MEL Institute Project (hereafter the MEL project). The MEL project was implemented in 2019 in Hong Kong and incorporated a certificate training course and a follow-up mentored practicum to build NGO practitioners’ EC at multidimensional levels (Ngai et al., 2022). An interdisciplinary team of experienced local and overseas trainers and mentors—including business, media, information technology, and social work experts—helped NGO practitioners acquire innovative knowledge and cutting-edge skills related to evaluations. The program’s certificate training course, which was implemented over the course of 2 months, systematically covered four interrelated areas: “service development and monitoring,” “resource and planning management,” “media and communications,” and “program evaluation and impact assessment.” Its aim was to help participants systematically acquire knowledge, skills, and attitudes that were conducive to successfully implementing the evaluation. Following the training workshops, a follow-up mentored practicum paired participants with mentors who coached them on ways to implement the acquired knowledge skills at their respective NGOs to effectively change the services of their organizations.

Participants were recruited from NGOs *via* email. Practitioners who indicated a willingness were interviewed and shortlisted to join the MEL project. They were also asked to invite other staff members with similar job duties who did not attend the MEL project but were interested in participating as members of the comparison group. Shortly thereafter, the participants (i.e., the training group) and their colleagues outside the project (i.e., the comparison group) were invited to be respondents. After the study participants were explicitly informed of the purpose, procedures, and related ethical information of this study, the research team obtained their signed consent to conduct the survey. All procedures were evaluated and approved by an ethics review committee prior to implementation.

A total of 439 NGO practitioners responded to the surveys before and after the MEL project, with 226 from the training group and 213 from the comparison group. This sample (*n* = 439) was used to develop and validate the proposed scale. Table 1 displays the profile of participants in the sample. The mean age of the participants was 38.24 years (SD = 8.64), more than half (64.5%) were female, most (59.0%) had a master’s degree, and nearly half (49.7%) were employed in social work positions, followed by management and administrative positions (29.2%). Notably, the profile of participants in this study is comparable to the existing profile of NGO practitioners in Hong Kong (Social Work Registration Board, 2022), which shows that most (57.95%) NGO practitioners in Hong Kong are aged from 30 to 49 (34.02% for the 30–39 age group, 23.93% for the 40–49 age group), more than half are female (68.92%), and most (67.53%) have a master’s degree. Accordingly, even though a nonprobability sampling strategy was adopted, sample characteristics of this study closely resemble those of the population of NGO practitioners in Hong Kong.

**Table 1:** Profile of participants in the sample.

Variables	Training group (*n* = 226)	Comparison group (*n* = 213)	Overall (*n* = 439)
Gender			
Male	37.2	33.8	35.5
Female	62.8	66.2	64.5
Educational level			
Sub-degree/diploma	0.9	9.9	5.2
Bachelor’s degree	28.8	41.3	34.9
Master’s degree	68.6	48.8	59.0
Doctoral degree	1.8	0.0	0.9
Job position			
Social worker	40.7	59.2	49.7
Healthcare professional	3.1	2.8	3
Manager/administrator	34.5	23.5	29.2
Social entrepreneur	3.5	0.5	2.1
Therapist	2.7	1.4	2.1
Others	15.5	12.7	14.1
Work areas (multiple choices)			
Child services	28.3	16.0	24.3
Youth services	28.3	23.5	28.2
Family services	23.5	16.4	21.8
Older adult services	28.3	19.2	26.0
Community development services	31.0	22.1	29.0
Services for individuals with disabilities	23.9	22.5	25.2
Educational services for disadvantaged groups	19.5	15.5	19.1
Social security services	17.7	4.2	12.1
Medical services	11.9	5.6	9.7
Services for ethnic minorities	6.2	2.8	5.0
Services for offenders and drug addicts	3.5	2.8	3.5
Employment support services	4.0	0.9	2.7
Age (years)			
Mean (*SD*)	39.52 (*8.32*)	36.89 (*8.78*)	38.24 (*8.64*)

### 3.2. Measures

The Evaluation Capacity Scale (ECS) assesses the capacity of NGO practitioners to conduct an effective evaluation. The development of the ECS followed five established procedures for scale development (Clark and Watson, 1995). First, after making reference to the existing literature, we defined EC as NGO practitioners’ ability to examine the effectiveness of their service programs, using results generated from the evaluation to further improve service quality and meet the needs of diverse stakeholders (Ngai et al., 2022). Second, we reviewed previous studies and proposed the following three dimensions to measure EC: (1) evaluation mindset, including an awareness of the significance of evaluation and relevant supporting resources, motivation to acquire and apply evaluation knowledge and tools, and competence (i.e., sufficient knowledge and skills) for evaluation (Bourgeois and Cousins, 2013; Morkel and Ramasobana, 2017); (2) evaluation implementation, namely the ability to engage in the full evaluation practice process, including conducting the needs assessment, formulating the evaluation plan, monitoring the process, and evaluating the final outcomes (Sonnichsen, 2000; Mitchell, 2017; Rossi et al., 2019); (3) evaluation communication, including the use of digital tools in the evaluation process and for evaluation result dissemination, as well as the necessary presentation skills for outcome/impact communication (Medina et al., 2015; Kettner et al., 2016; DeCorby-Watson et al., 2018). Third, we generated initial items to capture salient dimensions and their indicators specified above. This step involved deductive scale development approaches (Boateng et al., 2018), in which the aforementioned definitions for EC and its dimensions were used to guide item development (see the “Understanding Evaluation Capacity” section for more detailed definitions). This process generated 17 items that represent NGO practitioners’ EC. Fourth, a panel of 10 experts in evaluation or NGO development was invited to independently review the conciseness, clarity, and appropriateness of the proposed scale items. We considered their suggestions and feedback and revised the items accordingly. Fifth, we pilot tested the ECS with 10 practitioners from different NGOs. Their feedback on the clarity of the proposed items was incorporated into the revision of ECS. Since all respondents were professionals from the NGO sector who could read English proficiently, the ECS scale was developed using English and did not involve the English–Chinese translation procedure.

The ECS included a total of 17 items in a randomized order: seven items measuring evaluation mindset; six items for evaluation implementation; and four items assessing evaluation communication. Participants were asked to rate the number that reflects their actual conditions with a leading question: “*How much have you applied the following in your work?*” All items were measured on a five-point scale: 1 = *none*; 2 = *rather little*; 3 = *average*; 4 = *rather a lot*; 5 = *very much*.

First, the items related to evaluation mindset captured the extent of NGO practitioners’ familiarity with and interest in applying evaluation principles and practices (Bourgeois and Cousins, 2013). Sample items included “Having an understanding of program evaluation,” “Being confident applying program evaluation knowledge in your organization,” and “Appreciating program evaluation knowledge in informing service delivery.”

Second, items related to evaluation implementation referred to the extent to which NGO practitioners could conduct evaluations and use them within organizations (Taylor-Ritzler et al., 2013). Sample items included “Using more rigorous sampling procedures for data collection,” “Conducting problem analyses and needs assessments,” and “Practicing the effectiveness-based framework of monitoring, evaluation, and learning.”

Third, items related to evaluation communication focused on NGO practitioners’ ability to leverage conventional communication channels and digital tools to access and disseminate evaluation-related information. Sample items included “Using social media and the internet in participant recruitment and data collection,” “Using digital storytelling techniques in sharing evidence-based practices,” and “Conducting media and press interviews or conferences to build the brand, disseminate outcomes, and share impact.”

### 3.3. Data analysis

Several types of data analysis were used to develop and validate the psychometric properties of the ECS. Based on the baseline data collected from all 439 practitioners before the MEL project, exploratory factor analysis (EFA) and confirmatory factor analysis (CFA) were conducted. We randomly separated the entire sample into two subsamples for EFA and CFA, respectively. EFA with varimax rotation was performed with the first subsample (*n* = 237) to examine the ECS’ factor structure. To determine the number of factors, the “eigenvalue higher than 1″ criterion, scree plot, and amount of variance explained were considered. A minimum loading of 0.50 was used as the cutoff for an item to be part of a factor (Hair et al., 2010; Kim et al., 2016).

CFA, performed with another subsample (*n* = 202), was conducted to validate the scale’s latent structure generated from EFA. The model-fit indices were interpreted to determine the goodness of data-model fit based on a chi-square test (*χ*^2^), root mean square error of approximation (RMSEA), standardized root mean square residual (SRMR), and comparative fit index (CFI). RMSEA and SRMR values ranging from 0.05–0.09 indicated an acceptable model fit, with lower values indicating a better model fit (Kline, 2015; Erceg et al., 2020). CFI values greater than 0.90 were an acceptable model fit, and values exceeding 0.95 indicated a good model fit (Hu and Bentler, 1999; Kline, 2015). Furthermore, we anticipated that if convergent validity exists, the ECS subscales should converge, and the correlation between the three subscales should be significant and positive. Then, we tested the internal consistency reliability and construct validity of the ECS using the full sample.

Another means of evaluating the ECS validity was to determine the extent to which it is related to future outcomes by examining its predictive validity (Lin and Yao, 2014). As stated in the Introduction, one purpose of the ECS is to assess NGO practitioners’ ability to evaluate the program over time and measure the effectiveness of ECB activities. For this reason, MANCOVA was performed to test the predictive validity of the ECS, using baseline and follow-up data collected from 439 practitioners before and after the MEL project, with the status of having been trained serving as a fixed-factor independent variable, the scores for variables associated with EC post training as dependent variables, and the scores for EC variables prior to training and the sociodemographic variables (i.e., gender, age, level of education, and occupation) as covariates. All procedures in the data analysis were performed with IBM SPSS Statistics 25.0 and Mplus Version 8.

## 4. Results

### 4.1. Exploratory factor analysis

Before conducting the analysis, we performed the Kaiser–Meyer–Olkin test (KMO) to gage the suitability of the sample size for factorization. The KMO test yielded a value of 0.922, which confirmed the sample size of our study as sufficient for the factor analysis (Leech et al., 2007). Bartlett’s test of sphericity yielded a significant result—*χ*^2^ = 2,566.458, *p* < 0.001—which meant that the data were considered to have a multivariate normal distribution.

In EFA, three factors had eigenvalues greater than 1. In light of the abovementioned criteria, extracting three factors was deemed adequate. The total contribution of these factors—Factor 1, Factor 2, and Factor 3—to the common variance was 65.682%, with their individual contributions being 6.862%, 14.762%, and 44.058%, respectively. Those results indicated that the explained common variance was adequate for a multifactorial design (Costello and Osborne, 2005). Moreover, the factor loadings for Factors 1, 2, and 3 ranged from 0.623 to 0.741, from 0.570 to 0.793, and from 0.808 to 0.883, respectively. The factor loadings of the scale items exceeded the threshold value and were therefore deemed acceptable (see Table 2). Based on the theoretical foundation and meaning of the corresponding items, Factors 1, 2, and 3 were called *evaluation communication*, *evaluation implementation*, and *evaluation mindset*, respectively.

**Table 2:** Rotated factor loadings matrix from EFA (*n* = 237).

Items	Factors		
	1	2	3
EC 1: Using digital storytelling techniques in sharing evidence-based practice	0.674		
EC 2: Using social media and the Internet in participant recruitment and data collection	0.706		
EC 3: Conducting media and press interviews/conferences for brand building and outcome/impact dissemination	0.741		
EC 4: Using public presentation skills in sharing evidence-based practice	0.623		
EI 1: Avoiding ethics violation in data collection		0.603	
EI 2: Developing performance indicators for service development and monitoring		0.590	
EI 3: Using statistics in program evaluation		0.711	
EI 4: Practicing the effectiveness-based framework of monitoring, evaluation, and learning		0.570	
EI 5: Conducting problem analyses and needs assessment		0.793	
EI 6: Using more rigorous sampling procedures for data collection		0.669	
EM 1: Having an understanding of program evaluation			0.837
EM 2: Having awareness of available research tools and technological resources for conducting program evaluation			0.810
EM 3: Appreciating program evaluation knowledge in informing service delivery			0.881
EM 4: Being confident applying program evaluation knowledge in your organization			0.860
EM 5: Sharing program evaluation knowledge with colleagues			0.883
EM 6: Engaging in peer learning about program evaluation			0.879
EM 7: Having presentation skills in sharing program evaluation results			0.808

EC = Evaluation communication; EI = Evaluation implementation; EM = Evaluation mindset.

### 4.2. Confirmatory factor analysis and convergent validity

**Figure 1:**
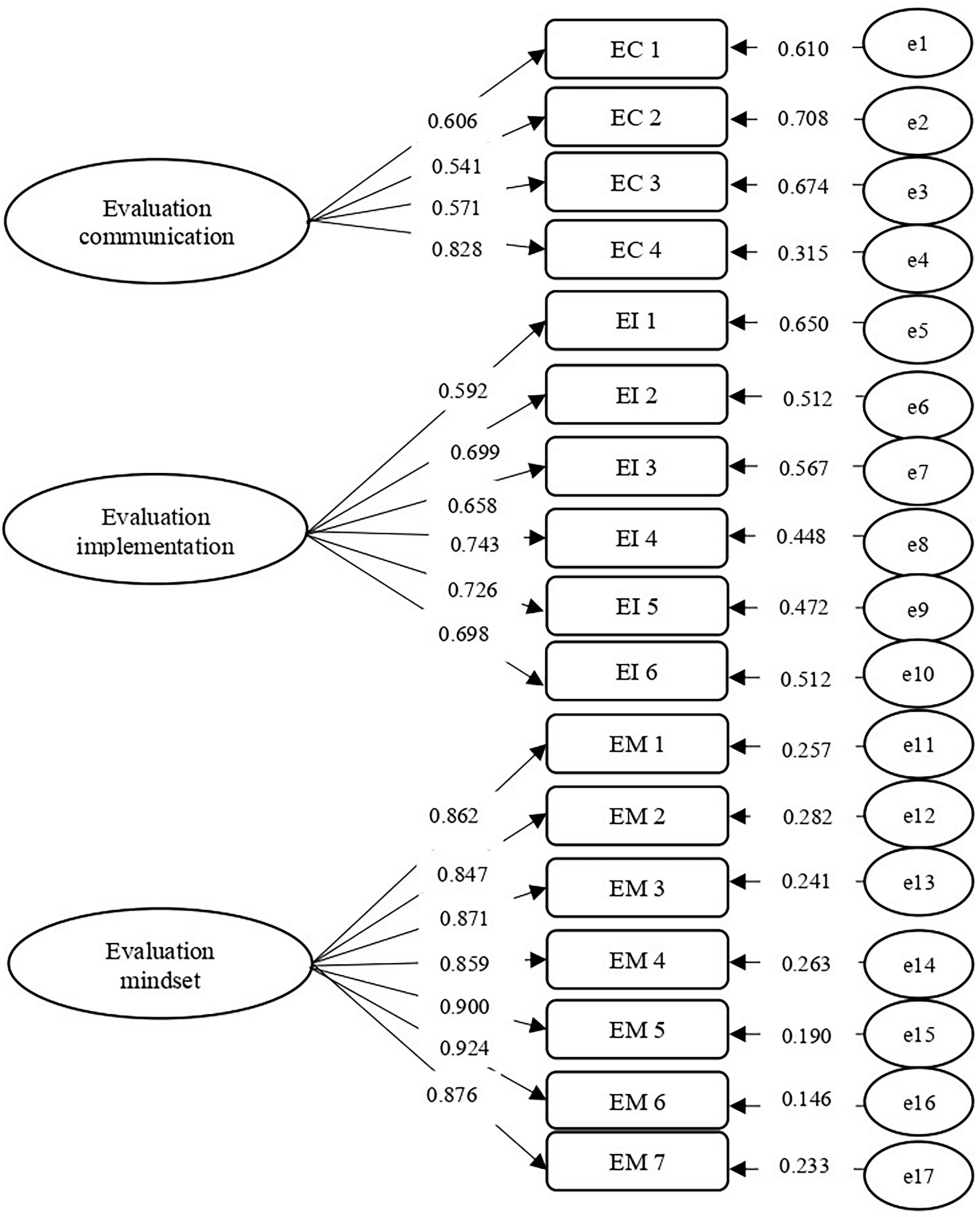
Validation of the Factor Structure with CFA (*n* = 202). All coefficients displayed in this figure were factor loadings that are statistically significant at *p* < 0.001 level. EC = Evaluation communication; EI = Evaluation implementation; EM = Evaluation mindset.

CFA, which was conducted to confirm the three-factor model obtained from the EFA, yielded acceptable model-fit indices of *χ*^2^ = 235.779, df = 116, *p* < 0.001, CFI = 0.948, RMSEA = 0.071, and SRMR = 0.050. As shown in Figure 1 and Table 3, all standardized factor loadings exceeded 0.50, which provided evidence supporting the scales’ construct validity. The factor loadings for *evaluation communication, evaluation implementation*, and *evaluation mindset* ranged from 0.541 to 0.828, from 0.592 to 0.743, and from 0.847 to 0.924, respectively.

**Table 3:** Factorial validity (*n* = 202).

Scale	Parameters of significance test				
	Items	Estimate	SE	Est./SE	*p*-Value
Factor 1: Evaluation communication (EC)	EC 1	0.606	0.052	11.636	***
	EC 2	0.541	0.057	9.429	***
	EC 3	0.571	0.055	10.393	***
	EC 4	0.828	0.034	24.149	***
Factor 2: Evaluation implementation (EI)	EI 1	0.592	0.052	11.463	***
	EI 2	0.699	0.042	16.483	***
	EI 3	0.658	0.046	14.263	***
	EI 4	0.743	0.038	19.322	***
	EI 5	0.726	0.039	18.401	***
	EI 6	0.698	0.042	16.429	***
Factor 3: Evaluation mindset (EM)	EM 1	0.862	0.020	43.655	***
	EM 2	0.847	0.022	39.088	***
	EM 3	0.871	0.019	46.557	***
	EM 4	0.859	0.020	42.624	***
	EM 5	0.900	0.015	59.018	***
	EM 6	0.924	0.012	74.777	***
	EM 7	0.876	0.018	48.058	***

***p < 0.001.

We further validated the three-factor model in subgroups according to gender (male vs. female), age (age ≤ median age of 38 years [younger] vs. age > median age of 38 years [older]), and level of education (level of education ≤ bachelor’s degree [lower level of education] vs. level of education > bachelor’s degree [higher level of education]). Table 4 presents the three-factor model’s goodness-of-fit in relation to each subgroup. The model fit indices were all acceptable: male (*n* = 156), CFI = 0.933, RMSEA = 0.083, SRMR = 0.053; female (*n* = 283), CFI = 0.922, RMSEA = 0.085, SRMR = 0.057; younger (*n* = 223), CFI = 0.945, RMSEA = 0.073, SRMR = 0.051; older (*n* = 216), CFI = 0.928, RMSEA = 0.081, SRMR = 0.058; lower level of education (*n* = 176), CFI = 0.946, RMSEA = 0.074, SRMR = 0.055; and higher level of education (*n* = 263), CFI = 0.931, RMSEA = 0.079, SRMR = 0.057. Results showed that the three-factor model of the ECS fit each subsample well.

**Table 4:** Factorial validation in subsamples categorized by gender, age, and education level.

	CFA of total sample model *n* = 439	Gender		Age		Education level	
		Male *n* = 156	Female *n* = 283	Younger *n* = 223	Older *n* = 216	Lower *n* = 176	Higher *n* = 263
Chi-square	235.779	239.723	351.372	254.279	281.111	228.997	306.276
Degrees of freedom	116	116	116	116	116	116	116
*P*-value	<0.001	<0.001	<0.001	<0.001	<0.001	<0.001	<0.001
CFI	0.948	0.933	0.922	0.945	0.928	0.946	0.931
RMSEA	0.071	0.083	0.085	0.073	0.081	0.074	0.079
SRMR	0.050	0.053	0.057	0.051	0.058	0.055	0.057

Moreover, as shown in Table 5, the correlation between evaluation mindset, evaluation implementation, and evaluation communication ranged from 0.445 to 0.612 (*p* < 0.001), which indicated a good convergent validity of the ECS.

**Table 5:** Correlations between evaluation mindset, evaluation implementation, and evaluation communication (*n* = 439).

	1	2	3
1. Evaluation mindset	1.00		
2. Evaluation implementation	0.612***	1.00	
3. Evaluation communication	0.450***	0.445***	1.00

***p < 0.001.

### 4.3. Reliability analysis

Cronbach’s alpha was used to estimate the scale’s internal consistency and that of each subscale. The Cronbach’s alphas for *evaluation mindset*, *evaluation implementation*, and *evaluation communication* at the pretest were 0.955, 0.821, and 0.747, respectively. At the posttest, the Cronbach’s alphas for *evaluation mindset*, *evaluation implementation*, and *evaluation communication* were 0.958, 0.868, and 0.826, respectively. The Cronbach’s alpha for the total scale was 0.918 for the pretest and 0.953 for the posttest. Notably, all results surpassed the standard, which states that a scale’s reliability is deemed sufficient if its Cronbach’s alpha exceeds 0.70, indicating satisfactory internal consistency (Hair et al., 2010; Gelashvili et al., 2021a,b).

### 4.4. Validity analysis

As for predictive validity, MANCOVA was conducted to test whether the ECS could detect any significant differences in NGO practitioners’ EC in the training group versus their counterparts in the comparison group. The statistically significant result (*F* = 29.5, *p* < 0.001) indicated differences between NGO practitioners in the training group and comparison group in posttest scores for evaluation mindset, evaluation implementation, and evaluation communication. By extension, the tests of between-subjects effects in MANCOVA were employed to determine which dependent variables had significantly changed. As shown in Table 6, *evaluation communication* (*F* = 11.984, *p* < 0.01), *evaluation implementation* (*F* = 15.059, *p* < 0.001), and *evaluation mindset* (*F* = 72.911, *p* < 0.001) all significantly increased in the training group after the MEL project. All of these results indicate the ECS’ predictive validity.

**Table 6:** Means and standard deviations of the main variables at pre- and post-training and the MANCOVA results.

Variables	Training group (*n* = 226)				Comparison group (*n* = 213)				MANCOVA		
	Mean		SD		Mean		SD				
	Pre	Post	Pre	Post	Pre	Post	Pre	Post	*F*	Sig	*η* ^2^
Evaluation communication	2.368	3.066	0.753	0.798	2.573	2.887	0.870	0.958	11.984	0.001	0.027
Evaluation implementation	2.786	3.357	0.719	0.675	2.924	3.144	0.763	0.836	15.059	<0.001	0.034
Evaluation mindset	2.288	3.399	0.894	0.642	2.010	2.694	0.958	1.061	72.911	<0.001	0.145

## 5. Discussion

Building NGO practitioners’ EC has become a prominent theme in the literature and is widely used by policy makers, funding agencies, and NGOs due to changing relationships between NGOs and their funding sources in the past two decades (Szczepanska, 2020). As policy makers emphasized devolution and decentralization, contracting and new opportunities emerged for NGOs (Suárez and Marshall, 2012), which spurred NGOs to improve capacity and obtain multilateral funding (Spolander et al., 2014; Szczepanska, 2020). In addition to pressure from funding agencies, the number of NGO management programs continues to grow, as does the need to strengthen the capacity of NGOs to fulfill multiple, increasingly complex roles (Preskill and Russ-Eft, 2015). Nevertheless, both theoretical and practical challenges persist, because little is known about how to assess NGO practitioners’ capacity to perform evaluations when few validated tools are available. Therefore, the development of the ECS in our study promises to be a valuable tool for NGO leaders, NGO practitioners, and researchers to understand and assess the capacity of NGO practitioners to conduct an effective evaluation of their service programs.

A two-phase analytic method involving the EFA and the CFA was used to investigate the empirical factor structure of the ECS. The EFA supported a three-factor structure comprising evaluation mindset, evaluation implementation, and evaluation communication, all of which were previously identified as important constructs when assessing EC (Preskill and Boyle, 2008; Bourgeois and Cousins, 2013; Taylor-Ritzler et al., 2013; Doherty et al., 2015; Medina et al., 2015; Kettner et al., 2016; DeCorby-Watson et al., 2018). The three-factor structure was also supported by the CFA and yielded results that indicated an acceptable model fit and factor loadings. Items for each factor clustered well, which suggests a strong interrelation between items, and the scale’s internal consistency was excellent. The model fit indices and factor loadings supported the construct validity of the ECS in the entire sample and in each subsample, and the MANCOVA results indicated a high predictive validity for the ECS. The current results suggest that the newly developed scale could be valid and reliable in the NGO context to assess practitioners’ Evaluation Capacity.

### 5.1. Theoretical implications

This study offers several theoretical contributions. There is widespread agreement that the evaluation field still lacks validated instruments to assess NGO practitioners’ EC (Nielsen et al., 2011; Suarez-Balcazar and Taylor-Ritzler, 2013). Our study fills the gap by developing a rigorous self-reporting measure, validating this scale with psychometric data, supplying empirical evidence of its validity and reliability, and making it available to NGO leaders, NGO practitioners, and researchers for assessing EC. Our findings echo the prior literature and strengthen a multidimensional conceptual foundation for understanding EC (e.g., Doherty et al., 2015). While existing measures largely focus on evaluators’ knowledge and skills (Medina et al., 2015; Morkel and Ramasobana, 2017), giving only slight attention to their mindset and communication, our study yielded unique empirical findings on a distinct three-factor structure of the ECS: evaluation mindset (seven items); evaluation implementation (six items); and evaluation communication (four items). The three factors included in the ECS offer an overview of how EC could be operationalized.

This study represents a first attempt to utilize an assessment tool (i.e., the ECS) for baseline and follow-up measurements of NGO practitioners’ existing capacity and the outcomes of an ECB program. First, as suggested earlier, despite other scales presented in the literature, a scarcity of validated measures existed, especially ones covering various domains of EC with operational items. Most of the existing instruments are checklists or guidelines (e.g., Brandon and Higa, 2004; Arnold, 2006), which are intrinsically general and unable to accurately assess NGO practitioners’ ability to evaluate the program over time or measure the effectiveness of ECB activities. In this context, it could be argued that the ECS—a rigorous instrument with demonstrated satisfactory convergent validity, internal consistency reliability, subgroup consistency, and predictive validity—could potentially be employed by NGO leaders, NGO practitioners, and researchers seeking to assess and build NGO practitioners’ EC. Moreover, while the 68-item scale derived by Taylor-Ritzler et al. (2013) has been one of the most widely used measures, its length presents a challenge to many respondents, the measuring domains are limited to the cognitive and behavioral domains, and they do not involve evaluation communication. For this reason, the ECS is not only a shorter user-friendly scale, but the development and validation thereof complements existing studies by providing empirical support for the applicability of the major dimensions suggested in the literature—evaluation mindset, evaluation implementation, and evaluation communication—in the context of NGOs (Preskill and Boyle, 2008; Bourgeois and Cousins, 2013; Doherty et al., 2015; Harman, 2019). Moreover, compared with Taylor-Ritzler et al.’s (2013) validation study, which recruited fewer participants (*n* = 169) with a lower proportion (11%) of frontline practitioners, our study included a larger sample (*n* = 439), a higher proportion (49.7%) of frontline practitioners and a diverse service area that ranged from community development services to services for offenders and drug addicts. Finally, we further validated the three-factor model in the subgroups according to gender, age, and level of education. This addition to both the scope and sample characteristics illustrates the applicability of the ECS for a variety of NGO practitioners.

### 5.2. Practical implications

Furthermore, this study offers significant practical implications. Recent funding constraints and the development of a contract culture have heightened the demand for increased accountability (Spolander et al., 2014). This pressure has led NGOs to emphasize the importance of effective capacity building to ensure a degree of accountability. As a consequence, there is broad recognition of the need to assess the capacity of NGO practitioners to implement evaluations related to the planning, designing, delivery, and evaluation of services rendered and ways to improve outcomes (Humphries et al., 2010). Meanwhile, improving NGO practitioners’ evaluation mindset has the potential to promote positive attitudes toward the use of evaluations in daily practices. Improving the manner in which evaluations are implemented would allow NGO practitioners to use their knowledge to perform rigorous evaluations. Notably, improving evaluation-related communication would benefit NGO practitioners who wish to use digital techniques and social media to access and disseminate the evaluation findings.

Our study regarding developing and validating the psychometric qualities of the ECS suggests that it may be used by NGO leaders, NGO practitioners, and researchers to assess the status of NGO practitioners’ EC. Using the ECS in practice will offer the potential to generate a useful understanding within NGOs of their practitioners’ capacity to perform evaluations, and the three subscales—evaluation communication, evaluation implementation, and evaluation mindset—can also help NGOs pinpoint areas that need improvement for better capacity building. The ECS can also be administered to NGO practitioners in a variety of service fields, in different age groups, and with different levels of education since our sample included NGO practitioners representing an array of service areas for disadvantaged groups in society. Overall, our findings provide evidence supporting the use of the ECS for assessing NGO practitioners’ current capacity and understanding how ECB activities may be tailored to effectively enhance their EC.

### 5.3. Limitations and future research

Despite these contributions and significance, our study revealed several limitations. The first limitation arose from the sampling procedure and sample size. Although we recruited practitioners from different types of NGOs and different service areas to increase participant diversity, we did not obtain a representative sample of Hong Kong NGO practitioners because the respondents were not randomly recruited. Hence, for future lines of research, we propose to adopt other sampling methods to recruit a larger population and a random sample to further examine the scale’s psychometric properties and to strengthen the evidence supporting the validity and reliability of the ECS. Second, although evaluation communication could be understood as one aspect of evaluation utilization (Kelly and Rogers, 2022), the current ECS does not fully assess evaluation utilization (e.g., using evaluation results for organizational decision-making). Another future research line could be adding evaluation utilization to our proposed multidimensional framework of EC and further validating the factor structure. In addition, future research could consider performing a correlational analysis with other criterion variables to provide additional information related to discriminant and concurrent validity. Finally, because our participants were limited to Hong Kong NGO practitioners, the findings may not be generalizable outside of this region; future studies with larger samples might overcome this limitation, and the ECS could thus be validated in regions with different sociocultural contexts (Gelashvili et al., 2021a,b).

## 6. Conclusion

This study developed the ECS, a self-reporting measure for NGO practitioners to assess their capacity to perform evaluations, and our findings provide empirical evidence supporting the use of the ECS across a wide spectrum of service fields in the Hong Kong context. The ECS taps into multiple domains of EC—evaluation mindset, evaluation implementation, and evaluation communication, and the initial assessment of its reliability and validity presented in this study yielded promising findings. The ECS also demonstrated satisfactory convergent validity, high internal consistency reliability and predictive validity, while its factor structure was supported in subgroups based on gender, age, and level of education. Ideally, this scale could be used as a measurement instrument to assess NGO practitioners’ EC as well as understanding their changes in these capacities following ECB activities. Future studies should address the delineated limitations of the present study by psychometrically testing the ECS and extending the generalization to other regions with the use of a larger representative sample.

## Data availability statement

The datasets generated and/or analyzed in the current study are not publicly available, as they contain information that could compromise the privacy of research participants. The data that support the findings of this study are available from the corresponding author upon reasonable request.

## Ethics statement

The studies involving human participants were reviewed and approved by Survey and Behavioral Research Ethics Committee of The Chinese University of Hong Kong. The patients/participants provided their written informed consent to participate in this study.

## Author contributions

SS-yN and C-kC: conceptualization. SS-yN, C-kC, LW, and SJ: methodology. SS-yN, C-kC, YL, LZ, LW, and SJ: validation. SS-yN, C-kC, LW, and LZ: formal analysis. SS-yN, C-kC, and YL: investigation. SS-yN: resources. SS-yN, LW, and LZ: data curation. SS-yN, C-kC, LZ, YL, and EN-hY: writing—original draft preparation. SS-yN, C-kC, LW, YL, LZ, and H-yT: writing—review and editing. SS-yN, C-kC, LZ, and EN-hY: visualization. SS-yN: supervision and funding acquisition. SS-yN and H-yT: project administration. All authors contributed to the article and approved the submitted version.

## Funding

This study is supported by The Hong Kong Jockey Club Charities Trust (2019–0022).

## Conflict of interest

The authors declare that the research was conducted in the absence of any commercial or financial relationships that could be construed as a potential conflict of interest.

## Publisher’s note

All claims expressed in this article are solely those of the authors and do not necessarily represent those of their affiliated organizations, or those of the publisher, the editors and the reviewers. Any product that may be evaluated in this article, or claim that may be made by its manufacturer, is not guaranteed or endorsed by the publisher.
