# Supplementary material for: Validating the evaluation capacity scale among practitioners in non-governmental organizations
Source: Front Psychol. 2022 Dec 23;13:1082313. doi: 10.3389/fpsyg.2022.1082313 (PMC9816125; doi:10.3389/fpsyg.2022.1082313)
Supplement: Supplementary file 1 [file Data_Sheet_1.PDF]

Supplementary Material

Evaluation Capacity Scale (ECS)

Please circle the number that reflects your conditions

A	**How much have you applied the following in your work?**	***None***	***Rather Little***	***Average***	***Rather a Lot***	***Very Much***
1.	Using digital storytelling techniques in sharing evidence-based practices	0	1	2	3	4
2.	Avoiding ethics violations in data collection	0	1	2	3	4
3.	Developing performance indicators for service development and monitoring	0	1	2	3	4
4.	Using social media and the Internet in participant recruitment and data collection	0	1	2	3	4
5.	Conducting media and press interviews or conferences to build the brand, disseminate outcomes, and share impact	0	1	2	3	4
6.	Using statistics in program evaluation	0	1	2	3	4
7.	Using public presentation skills in sharing evidence-based practices	0	1	2	3	4
8.	Practicing the effectiveness-based framework of monitoring, evaluation, and learning	0	1	2	3	4
9.	Conducting problem analyses and needs assessments	0	1	2	3	4
10.	Using more rigorous sampling procedures for data collection	0	1	2	3	4
11.	Being confident in applying MEL knowledge and skills in your services	0	1	2	3	4
12.	Sharing program evaluation knowledge with colleagues	0	1	2	3	4
13.	Appreciating program evaluation knowledge in informing service delivery	0	1	2	3	4
14.	Engaging in peer learning about program evaluation	0	1	2	3	4
15.	Having an understanding of program evaluation	0	1	2	3	4
16.	Having presentation skills in sharing program evaluation results	0	1	2	3	4
17.	Having awareness of available research tools and technological resources for conducting program evaluation	0	1	2	3	4

B	Personal Information	
1.	Age	_______ years
2.	Gender	1. Male 2. Female
3.	What is your highest education?	1. Sub-degree or diploma<br>2. Undergraduate<br>3. Master’s<br>4. Doctoral
4.	What is your job position?	1. Social worker<br>2. Health care professional<br>3. Manager/administrator<br>4. Social entrepreneur<br>5. Therapist (e.g. occupational therapist, physiotherapist)<br>6. Others:________________
5.	What is the major work area? (You may circle more than one)	1. Children<br>2. Community<br>3. Education<br>4. Elderly<br>5. Ethnic minority/migration<br>6. Family<br>7. Labor<br>8. Medical/nursing<br>9. Offender/addict<br>10. Physical/mental problem rehabilitation<br>11. Youth<br>12. Welfare/security<br>13. Others: __________________
